# Neurology: Triple Threat Activates Neurons

**Published:** 2005-06

**Authors:** Carol Potera

Scientists from the Marine Biological Laboratory in Woods Hole, Massachusetts, have reported on a potentially sinister synergy, showing that a combination of three common pollutants—bromoform, chloroform, and tetrachloroethylene—alters nerve cell development, whereas the toxicants alone or in pairs do not. The discovery is an intriguing first step toward understanding whether this trio of pollutants is linked to neurological disorders such as autism.

Carol Reinisch, an expert in chemical-induced neurotoxicity, had read in the scientific literature about the contamination of municipal drinking water in Brick Township, New Jersey, and its possible connection to higher autism rates in local children. Chemical wastes dumped at the town’s landfill over the years had contaminated nearby wells with bromoform, chloroform, and tetrachloroethylene, and in 1983, the U.S. Environmental Protection Agency declared the landfill a Superfund site. In the 1990s, autism rates in the town started rising, and researchers from the Centers for Disease Control and the Agency for Toxic Substances and Disease Registry began to investigate in 1998. Although the incidence of autism was twice the national average, the federal scientists concluded in 2000 that the levels of individual well water contaminants were too low to adversely impact children’s health.

Reinisch wondered whether the synergistic effect of the chemicals would tell a different story. Her lab was already using a surf clam (*Spisula solidissima*) embryo model to assess how polychlorinated biphenyls affect embryonic neuronal development. The transparency of the embryos and the fact that most basic molecular processes involved in early development are conserved across species make the surf clam a good model for such studies. She and her colleagues began studying the three well-water contaminants in combination.

When tested alone or in pairs, the toxicants produced no significant changes, even at levels 1,000 times those in the mixture. But the trio acted synergistically to upregulate a regulatory subunit of cAMP-dependent protein kinase, a ubiquitous protein involved in neurologic pathways and a key regulator of neuronal growth in the clam embryo model. The clam embryos also showed increased cilia movement. The study appears in the January 2005 issue of *Environmental Toxicology and Pharmacology*.

“The fact that several events are speeded up is abnormal,” says Reinisch. Coauthor Jill Kreiling, a developmental biologist, adds, “We found something unusual going on neurologically, but we cannot say this is causing autism.”

Now the team is testing the mixture in zebrafish embryos, and their preliminary results parallel those for clam embryos. They hope others will undertake experiments in mice, rats, and higher mammals in order to confirm the association.

Studying mixtures of toxicants yields a more accurate picture of how contaminants work in the environment. “Most risk assessments look at single chemicals acting on single target organs with single outcomes, but that’s not the way [exposures] work in nature,” says Nigel Fields, a research program manager at the Environmental Protection Agency, which funded Reinisch’s project.

## Figures and Tables

**Figure:**
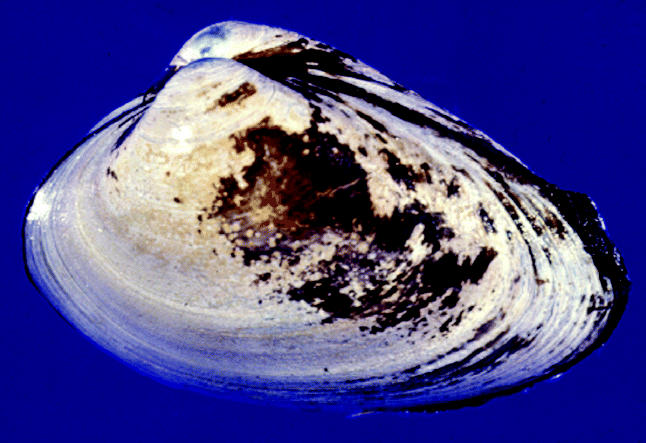
**Brain teaser.** The embryo of the surf clam yields intriguing clues to a potential neuronal threat.

